# Associations of Circulating Irisin Concentrations With Cardiometabolic Risk Factors Among Children Vary by Physical Activity or Sedentary Time Levels

**DOI:** 10.3389/fendo.2019.00549

**Published:** 2019-08-14

**Authors:** Li Cai, Minyi Tan, Weiqing Tan, Xia Zeng, Nianqing Wan, Stephen Heung-sang Wong, John O'Reilly, Fenghua Sun, Jiewen Yang, Yajun Chen

**Affiliations:** ^1^Department of Maternal and Child Health, School of Public Health, Sun Yat-sen University, Guangzhou, China; ^2^Health Promotion Centre for Primary and Secondary Schools of Guangzhou Municipality, Guangzhou, China; ^3^Department of Sports Science and Physical Education, The Chinese University of Hong Kong, Hong Kong, China; ^4^Department of Health and Physical Education, The Education University of Hong Kong, Hong Kong, China

**Keywords:** irisin, cardiometabolic risk factors, children, physical activity, sedentary time

## Abstract

Whether irisin concentrations are associated with physical activity (PA) and sedentary time (ST) remains unknown. The role of irisin on cardiometabolic health among children has been contradictory and scarce. This study aimed to examine associations of PA and ST with irisin concentrations and relationships between irisin concentrations and cardiometabolic parameters among children. Additionally, we assessed the interaction between PA or ST and irisin concentrations on cardiometabolic parameters. Basing on a cross-sectional survey of 3,651 general children aged 7–12 years, 575 with different self-reported PA (moderate-vigorous intensity PA ≥ 60 min/day or <150 min/week) and ST (gender-, age-specific ST ≥ 75% or <25% percentile) levels were selected. PA and ST were assessed by the validated international physical activity questionnaires. Fasting blood glucose and lipid profile levels were measured with standard methods by biochemistry analyzer. Plasma irisin concentrations were measured by ELISA. The associations of PA and ST with circulating irisin concentrations were examined by linear regression. Linear regression analysis was also used to estimate associations of circulating irisin concentrations with cardiometabolic variables. Interactions between PA or ST and irisin concentrations on cardiometabolic parameters were calculated using multiple linear regression models with dichotomized factors (low PA and high PA; low ST and high ST). No significant association was observed between circulating irisin concentrations and habitual PA or ST. Irisin concentrations were negatively associated with body mass index (BMI) (β = −0.220), BMI z-score (β = −0.098), waist circumference (β = −0.621), diastolic blood pressure (DBP) (β = −0.561), and triglyceride (β = −0.019) in low PA subgroup, and negatively related to fasting blood glucose (β = −0.040) among high PA subgroup. Moreover, irisin concentrations were negatively associated with BMI (β = −0.157) and fasting blood glucose (β = −0.026) only in high ST subgroup (all *P* < 0.05). We also observed a significant interaction between PA and irisin concentrations on BMI (*P*_interaction_ = 0.0350), BMI z-score (*P*_interaction_ = 0.0173), and DBP (*P*_interaction_ = 0.0068). In summary, irisin concentrations were not associated with habitual PA or ST in children. The negative associations of irisin concentrations with BMI, BMI z-score, and DBP were found only among children being inactive, implying that irisin may contribute to an improvement in health, especially among children with unhealthy lifestyles.

## Introduction

There has been a remarkable worldwide increase in the prevalence of cardiometabolic risk factors among children, including obesity, hypertension, hyperglycemia, and dyslipidemia (1–3). Evidence suggests that cardiometabolic risk factors in childhood potentially play a crucial role in influencing later susceptibility to cardiometabolic disease and portend higher mortality (4, 5).

Irisin, a newly identified myokine, has been suggested to regulate energy metabolism (6). It is speculated that irisin induces the browning of white adipose tissue and has a beneficial impact on energy homeostasis (6). Therefore, irisin administration has been suggested to potentially serve as a therapeutic target for cardiometabolic disorders in the future. Some studies have put emphasis on associations of circulating irisin concentrations with cardiometabolic outcomes but conflicting conclusions have been observed (7–10). The controversial findings across studies may be linked with the discrepancy of various characteristics among study populations, such as age, sex, body mass index (BMI) status, diet, and physical activity (PA) levels (7, 11, 12). Research from Spain indicated that irisin concentrations were positively associated with metabolic risk factors among inactive adults, but not among active subjects (12). It is of interest whether associations between circulating irisin concentrations and cardiometabolic parameters are differentially regulated by PA levels. Although evidence has raised concerns about the role of irisin on cardiometabolic improvement, irisin has not been widely studied among children. Variations in different growth development stages may affect the role of irisin. Generalizing findings from adults to children may not be appropriate.

In addition, it has been suggested that increased PA and decreased sedentary time (ST) are important contributors in the protection against cardiometabolic risk factors via myokine released from skeletal muscle (13, 14), and both variables may be independently associated with cardiometabolic risk factors (15). However, the effects of PA on circulating irisin concentrations in humans remain controversial. Several studies have indicated that circulating irisin concentrations were increased in response to acute exercise or habitual PA levels (16, 17), whereas others failed to confirm a significant association between PA and irisin concentrations (18–20). Furthermore, despite the epidemic of ST, to our knowledge no data exist on the relationship between ST and irisin concentrations.

Given this partially unclear situation, the study aimed to (1) examine associations of PA and ST with circulating irisin concentrations and (2) investigate the relationship between irisin concentrations and cardiometabolic parameters among children. (3) In addition, we assessed the interaction between PA or ST and irisin concentrations on cardiometabolic parameters.

## Materials and Methods

### Study Design and Participants

This study was a cross-sectional survey based on a National Natural Science Foundation project of China (Registration number: NCT 03582709), which was conducted between 2017 and 2018. The project had been approved by the Ethical Committee of Sun Yat-sen University and all the participants and their parents signed the informed consents voluntarily prior to participation in the study. In brief, one primary school was randomly chosen from each of the selected 5 different districts in Guangzhou. All the students from these schools were invited to participate in our study. Subjects with significant congenital anomalies or severe handicaps, or prescription medication that might affect endocrine function were excluded. Eventually, 3,651 subjects aged 7–12 years with available PA and ST information as well as blood samples were involved for further analysis.

**Figure 1:**
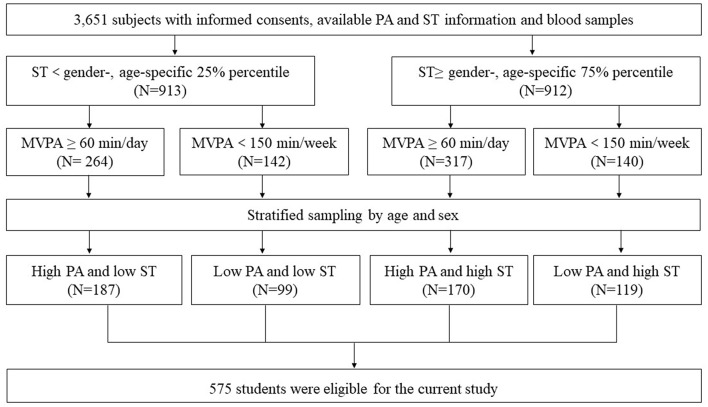
The sampling process.

According to the information on PA and ST obtained by the validated international physical activity questionnaire (IPAQ) (21), subjects with ST equal to or above the gender-, age-specific 75% percentile were selected as the high ST subgroup, while those with ST below the gender-, age-specific 25% percentile were selected as the low ST subgroup. Among each ST subgroup, subjects with moderate-vigorous physical activity (MVPA) ≥ 60 min/day were assigned to the high PA subgroup and those with MVPA <150 min/week were assigned to the low PA subgroup, respectively (22). Subsequently, 863 subjects who met the PA and ST criterion mentioned above were assigned into 4 groups with different ST and PA levels. After stratified sampling by age and sex, 575 children were eligible for the current study (Figure 1).

### Measurements

#### Physical Activity and Sedentary Time Measurements

Information on PA, ST, food intake, and demography was collected by a validated questionnaire, which was filled out by the participants together with their parents. To determine the habitual PA and ST levels, IPAQ (21) was used to assess the frequency and duration of vigorous activity (VPA), moderate activity (MPA), walking, and ST in the last 7 days. VPA referred to activities that take hard physical effort and make you breathe much harder than normal. MPA referred to activities that take moderate physical effort and make you breathe somewhat harder than normal. VPA (min/day) and MPA (min/day) were continuous variables which could be calculated directly from the questionnaire. MVPA was the sum of MPA and VPA. ST covered time which spent in homework, television viewing, and computer using, but not in class or motor vehicle. Only sustained activities of more than 10 min would be noted. To determine participants' food intakes, the frequency and amount of fruit, vegetable, grain, fish, meat, fried food, milk, and sugar-sweetened beverage intake in the last 7 days was collected. Average daily intakes of these foods were calculated as the following formula: average daily intake = [days × (amount in each of those days)]/7.

#### Anthropometric Measurements

Anthropometric variables were measured by professional doctors, following a standard protocol. Participants were asked to wear light clothing and no shoes when measuring height, weight, and waist circumference (WC). Height was measured to the nearest millimeter by a portable stadiometer. Weight was measured to the nearest 0.1 kg by a lever type weight scale. WC was measured to the nearest millimeter at the level of 1 cm above umbilicus with a steel tape. BMI was calculated as body weight in kilograms divided by height in meters squared (kg/m^2^). BMI z-score were calculated according to the age-, gender-specific standards from WHO (5–19 years) (23). Waist height ratio (WHtR) was calculated as WC in centimeters divided by height in centimeters.

Blood pressure was measured on the right arm by a validated electronic sphygmomanometer. Participants were required to stay calm for at least 5 min prior to measuring. The cuff was placed 2 cm above the crease of elbow and the size was depended on the mid upper arm circumference. Systolic blood pressure (SBP) and diastolic blood pressure (DBP) were recorded. Blood pressure was measured 3 times with 1-min intervals. The average of these three blood pressure recordings was used in the statistical analysis.

#### Biochemical Analysis

Blood samples were collected via venipuncture from the median cubital vein by qualified nurses at school in the morning, after a 12-h overnight fast. Participants were asked to rest and remain quiet for at least 5 min before the blood draw. Blood samples were immediately centrifuged at 3,000 rpm for 15 min. Plasma samples were separated as soon as possible and stored at −80°C for further assay. Fasting blood glucose levels were measured by the glucose oxidase method (HITACHI 7180 automated biochemistry analyzer, Tokyo, Japan). Concentrations of total cholesterol, triglycerides, high-density lipoprotein (HDL-C) and low-density lipoprotein (LDL-C) were determined by enzymatic assays (HITACHI 7180 automated biochemistry analyzer, Tokyo, Japan). Plasma irisin concentrations were measured with a specific commercial enzyme-linked immunosorbent assay (ELISA) kit (catalog no. EK-067-29; Phoenix Pharmaceuticals, CA, USA) with intra- and inter-assay variations of <10 and <15%, respectively.

#### Statistical Analysis

Statistical analyses were conducted using the SPSS software version 22.0. Continuous variables were expressed as mean ± standard deviation (SD). Values were presented as percentages for categorical variables. Differences in characteristics between groups were tested by analysis of variance (ANOVA) or chi-square test. Variables with non-normal distributions were log-transformed before analysis. The associations of PA and ST with circulating irisin concentrations were examined by linear regression. Model 1 was adjusted for age and sex. Model 2 was further adjusted for parental educational levels, family income, and food intake. Linear regression analysis was also used to estimate associations of circulating irisin concentrations with anthropometric and metabolic variables, adjusting for age, sex, parental educational levels, family income, PA (min/day), ST (min/day), and food intake. Interactions between PA or ST and circulating irisin concentrations on cardiometabolic parameters were calculated using multiple linear regression models with dichotomized factors (low PA and high PA; low ST and high ST), adjusting for age, sex, parental educational levels, family income, PA (min/day), ST (min/day), and food intake. A two-sided of *P*-value below 0.05 was considered as statistically significant.

## Results

The general characteristics of subjects are shown in Table 1. A total of 575 students were included in the study, of whom 32.5% with low ST and high PA level, 17.2% with low ST and low PA level, 29.6% with high ST and high PA level, and 20.7% with high ST and low PA level. Subjects had a mean age of 9.19 ± 1.61 years. Individuals with high ST and high PA level had higher body height and triglyceride levels compared with their counterparts. Circulating irisin concentrations and other cardiometabolic parameters were not significantly different among groups.

**Table 1:** Characteristics of subjects by physical activity and sedentary time levels.

	**Low ST level**		**High ST level**		**Total**	***P* value**
	**High PA level**	**Low PA level**	**High PA level**	**Low PA level**		
*N* (%)	187 (32.5)	99 (17.2)	170 (29.6)	119 (20.7)	575	
Age (year)	9.22 ± 1.62	8.78 ± 1.59	9.30 ± 1.59	9.35 ± 1.64	9.19 ± 1.61	**0.0363**
Sex (boy, %)	50.80	47.47	52.94	52.10	51.13	0.8482
Paternal educational level						**0.0014**
Primary or less (%)	0.00	0.00	0.60	0.00	0.18	
Junior or senior high school (%)	19.13	15.31	34.52	31.93	25.70	
College degree or above (%)	80.87	84.69	64.88	68.07	74.12	
Maternal educational level						**0.0006**
Primary or less (%)	2.19	1.02	2.98	0.00	1.76	
Junior or senior high school (%)	15.30	19.39	29.76	35.29	24.47	
College degree or above (%)	82.51	79.59	67.26	64.71	73.77	
Family income						**0.0016**
5,000 or below (%)	19.79	13.13	27.06	31.93	23.30	
5,000~12,000 (%)	45.99	46.46	42.94	28.57	41.57	
12,000 or above (%)	22.99	28.28	17.06	18.49	21.22	
Not clear (%)	11.23	12.12	12.94	21.01	13.91	
Height (cm)	139.72 ± 12.89	136.41 ± 10.23	141.36 ± 12.26	139.56 ± 11.63	139.60 ± 12.10	**0.0166**
Weight (kg)	33.70 ± 11.22	32.05 ± 9.18	35.22 ± 11.51	35.11 ± 10.60	34.14 ± 10.90	0.0956
BMI (kg/m^2^)	16.81 ± 3.13	16.97 ± 3.15	17.20 ± 3.10	17.69 ± 3.25	17.13 ± 3.16	0.1179
BMI z-score	−0.1 ± 1.35	0.09 ± 1.41	0.08 ± 1.32	0.30 ± 1.46	0.06 ± 1.39	0.1135
WC (cm)	58.89 ± 9.37	58.99 ± 9.29	60.12 ± 9.14	60.82 ± 10.80	59.66 ± 9.62	0.3025
WHtR	0.42 ± 0.05	0.43 ± 0.06	0.42 ± 0.05	0.44 ± 0.07	0.43 ± 0.05	0.0766
SBP (mmHg)	102.38 ± 16.25	100.74 ± 10.10	101.78 ± 11.32	102.48 ± 11.86	101.91 ± 13.04	0.7434
DBP (mmHg)	64.11 ± 8.50	64.43 ± 8.12	63.98 ± 8.23	66.18 ± 8.76	64.54 ± 8.44	0.1326
Fasting blood glucose (mmol/L)	5.05 ± 1.00	4.91 ± 0.40	5.11 ± 0.36	5.04 ± 0.41	5.04 ± 0.65	0.1179
Total cholesterol (mmol/L)	4.30 ± 0.74	4.45 ± 0.85	4.40 ± 0.80	4.37 ± 0.65	4.37 ± 0.76	0.4611
Triglyceride (mmol/L)	0.77 ± 0.29	0.77 ± 0.30	0.87 ± 0.42	0.87 ± 0.33	0.82 ± 0.35	**0.0094**
HDL-C (mmol/L)	1.55 ± 0.32	1.59 ± 0.35	1.54 ± 0.30	1.48 ± 0.30	1.54 ± 0.31	0.0812
LDL-C (mmol/L)	2.57 ± 0.71	2.71 ± 0.82	2.61 ± 0.72	2.69 ± 0.63	2.63 ± 0.72	0.3182
MPA (min/day)	51.99 ± 42.50	7.28 ± 6.32	62.88 ± 55.84	6.51 ± 6.00	38.06 ± 46.15	**<0.0001**
VPA (min/day)	46.35 ± 33.69	5.02 ± 5.71	56.63 ± 41.56	5.45 ± 5.61	33.77 ± 37.44	**<0.0001**
MVPA (min/day)	98.34 ± 46.80	12.30 ± 7.51	119.51 ± 74.55	11.96 ± 8.04	71.84 ± 67.97	**<0.0001**
ST (min/day)	11.70 ± 3.12	11.24 ± 3.18	38.81 ± 11.40	38.72 ± 10.19	25.19 ± 15,81	**<0.0001**
Irisin (ng/ml)	9.41 ± 2.51	9.19 ± 2.48	9.31 ± 2.24	9.25 ± 2.66	9.31 ± 2.45	0.8862

*BMI, body mass index; WC, waist circumference; WHtR, waist height ratio; SBP, systolic blood pressure; DBP, diastolic blood pressure; HDL-C, high-density lipoprotein; LDL-C, low-density lipoprotein; MPA, moderate physical activity; VPA, vigorous physical activity; MVPA, moderate-vigorous physical activity; ST, sedentary time. Values are mean ± SD or proportion. High PA subgroup includes subjects with moderate-vigorous physical activity ≥ 60 min/day; low PA subgroup includes subjects with moderate-vigorous physical activity <150 min/week. High ST subgroup includes subjects with sedentary time ≥ gender-, age-specific P_75_. Low ST subgroup includes subjects with sedentary time < gender-, age-specific P_25_. Differences of continuous variables between groups were tested by ANOVA. Differences of categorical variables between groups were tested by chi square test. Bold values indicate statistically significant*.

The associations of PA and ST with circulating irisin concentrations are presented in Table 2. After adjustments for covariates, there was no significant association between circulating irisin concentrations and PA. In addition, ST was not significantly related to irisin concentrations as well. Stratified analysis by gender showed similar results in both boys and girls (Table S1).

**Table 2:** Associations of physical activity and sedentary time with subjects' irisin concentrations.

	**Model 1**^a^		**Model 2**^b^	
	**Coefficient**	***P***	**Coefficient**	***P***
VPA (min/day)	0.00306	0.2631	0.00287	0.3381
MPA (min/day)	−0.00009526	0.9656	0.00109	0.6568
MVPA (min/day)	0.00088048	0.5581	0.00145	0.3948
ST (min/day)	−0.00077497	0.4047	−0.00063676	0.5210

*VPA, vigorous physical activity; MPA, moderate physical activity; MVPA, moderate-vigorous physical activity; ST, sedentary time*.

a *Model 1 was adjusted for age and sex*.

b *Model 2 was adjusted for variables in Model 1, parental educational levels, family income, and food intake*.

Table 3 presents the associations of subjects' circulating irisin concentrations and cardiometabolic parameters in the overall group. Circulating irisin concentrations were negatively associated with BMI (β = −0.12061, *P* = 0.0194), BMI z-score (β = −0.04978, *P* = 0.0349), WC (β = −0.36048, *P* = 0.0195), and fasting blood glucose levels (β = −0.03142, *P* = 0.0046), when adjusting for age and sex. The negative correlation between circulating irisin concentrations and fasting glucose levels remained significant in Model 2 (β = −0.03513, *P* = 0.0051). However, the associations of circulating irisin concentrations with BMI, BMI z-score, and WC became non-significant after further adjustment for other potential confounders. No significant association was found between circulating irisin concentrations and blood pressure as well as lipid profiles in the overall subjects. When stratified by gender, a negative association between irisin concentration and fasting glucose was observed only in boys (Table S2).

**Table 3:** Associations of subjects' irisin concentrations with cardiometabolic parameters.

	**Model 1^a^**		**Model 2^b^**	
	**Coefficient**	***P***	**Coefficient**	***P***
**Adiposity**				
BMI (kg/m^2^)	−0.12061	**0.0194**	−0.08940	0.1007
BMI z-score	−0.04978	**0.0349**	−0.03451	0.1649
WC (cm)	−0.36048	**0.0195**	−0.24069	0.1409
WHtR	−0.00167	0.0743	−0.00090	0.3706
**Blood pressure**				
SBP (mmHg)	−0.14307	0.5122	−0.06555	0.7634
DBP (mmHg)	−0.10277	0.4808	−0.10599	0.4729
**Glycemic parameters**				
Fasting glucose (mmol/L)	–**0.03142**	**0.0046**	–**0.03513**	**0.0051**
**Lipid parameters**				
Total cholesterol (mmol/L)	−0.02407	0.0643	−0.01828	0.2081
Triglyceride (mmol/L)	−0.00928	0.1163	−0.00849	0.1798
HDL-C (mmol/L)	−0.00272	0.6142	−0.00149	0.7992
LDL-C (mmol/L)	−0.01602	0.1914	−0.01047	0.4409

*BMI, body mass index; WC, waist circumference; WHtR, waist height ratio; SBP, systolic blood pressure; DBP, diastolic blood pressure; HDL-C, high-density lipoprotein; LDL-C, low-density lipoprotein*.

a *Model 1 was adjusted for age and sex*.

b *Model 2 was adjusted for variables in Model 1, parental educational levels, family income, physical activity (min/day), sedentary time (min/day), and food intake*.

*Bold values indicate statistically significant*.

To explore the interactions of PA or ST and circulating irisin concentrations on cardiometabolic parameters, we divided subjects into 2 subgroups by PA or ST levels (Table 4). When stratified by PA status, we found that higher circulating irisin concentrations were associated with lower BMI (β = −0.220, *P* = 0.0164), BMI z-score (β = −0.098, *P* = 0.0177), WC (β = −0.621, *P* = 0.0362), DBP (β = −0.561, *P* = 0.0222), and triglyceride levels (β = −0.019, *P* = 0.0428) only in the low PA subgroup. In addition, a negative association was observed between circulating irisin concentrations and fasting blood glucose among individuals with high PA status (β = −0.040, *P* = 0.0411). Significant interactions were observed between circulating irisin concentrations and PA levels on BMI (*P* = 0.0350 for interaction), BMI z-score (*P* = 0.0173 for interaction), and DBP (*P* = 0.0068 for interaction). Moreover, the interaction between circulating irisin concentrations and PA levels on WC was at the edge of significance (*P* = 0.0533 for interaction). Analysis stratified by ST levels was also conducted. Circulating irisin concentrations were inversely related to BMI (β = −0.157, *P* = 0.0346) and fasting blood glucose levels (β = −0.026, *P* = 0.0082) only in high ST subgroup. No significant interaction was observed between circulating irisin concentrations and ST levels.

**Table 4:** Associations of irisin concentrations with cardiometabolic parameters and interactions between irisin concentrations and PA/ST status.

	PA^*^Irisin			ST^*^Irisin		
	**Low PA subgroup**	**High PA subgroup**	***P*_**interaction**_**	**Low ST subgroup**	**High ST subgroup**	***P*_**interaction**_**
**Adiposity**						
BMI (kg/m^2^)	**−0.220 (−0.399**, −0.040)^*^	0.008 (**–**0.125, 0.141)	**0.0350**	**–**0.028 (**–**0.189, 0.134)	**−0.157 (−0.302**, −0.011)^*^	0.1400
BMI z-score	**−0.098 (−0.179**, −0.017)^*^	0.015 (**–**0.046, 0.076)	**0.0173**	**–**0.010 (**–**0.084, 0.065)	**–**0.061 (**–**0.127, 0.004)^#^	0.1723
WC (cm)	**−0.621 (−1.201**, −0.040)^*^	0.004 (**–**0.368, 0.375)	0.0533	**–**0.091 (**–**0.554, 0.373)	**–**0.392 (**–**0.849, 0.066)^#^	0.2367
WHtR	**–**0.003 (**–**0.006, 0.000)	0.0003 (**–**0.002, 0.003)	0.1498	0.000 (**–**0.003. 0.003)	**–**0.002 (**–**0.004, 0.001)	0.4119
**Blood pressure**						
SBP (mmHg)	**–**0.450 (**–**1.069, 0.169)	0.233 (**–**0.353, 0.819)	0.1429	0.015 (**–**0.676, 0.705)	**–**0.241 (**–**0.786, 0.303)	0.5665
DBP (mmHg)	**−0.561 (−1.040**, −0.081)^*^	0.239 (**–**0.127, 0.605)	**0.0068**	0.067 (**–**0.347, 0.481)	**–**0.282 (**–**0.701, 0.137)	0.2110
**Glycemic parameters**						
Fasting glucose (mmol/L)	**–**0.019 (**–**0.042, 0.003)^#^	**−0.040 (−0.079**, −0.002)^*^	0.7111	**–**0.042 (**–**0.089, 0.004)^#^	**−0.026 (−0.045**, −0.007)^**^	0.8514
**Lipid parameters**						
Total cholesterol (mmol/L)	**–**0.005 (**–**0.048, 0.039)	**–**0.026 (**–**0.064, 0.012)	0.5499	**–**0.009 (**–**0.052, 0.033)	**–**0.021 (**–**0.059, 0.017)	0.4933
Triglyceride (mmol/L)	**−0.019 (−0.038**, −0.001)^*^	**–**0.002 (**–**0.019, 0.015)	0.2233	**–**0.002 (**–**0.018, 0.013)	**–**0.013 (**–**0.033, 0.007)	0.1949
HDL-C (mmol/L)	0.014 (**–**0.004, 0.032)	**–**0.010 (**–**0.025, 0.005)	0.0828	0.002 (**–**0.016, 0.019)	**–**0.003 (**–**0.019, 0.012)	0.6246
LDL-C (mmol/L)	−0.013 (−0.054, 0.028)	−0.009 (−0.045, 0.026)	0.8936	−0.013 (−0.054, 0.028)	−0.005(−0.040, 0.031)	0.9671

BMI, body mass index; WC, waist circumference; WHtR, waist height ratio; SBP, systolic blood pressure; DBP, diastolic blood pressure; HDL-C, high-density lipoprotein; LDL-C, low-density lipoprotein; PA, physical activity; ST, sedentary time. High PA subgroup includes subjects with moderate-vigorous physical activity≥60 min/day; low PA subgroup includes subjects with moderate-vigorous physical activity <150 min/week. High ST subgroup includes subjects with sedentary time ≥ gender-, age-specific P_75_. Low ST subgroup includes subjects with sedentary time < gender-, age-specific P_25_. Models were adjusted for age, sex, parental educational levels, family income, physical activity (min/day), sedentary time (min/day), and food intake;

* *P < 0.05*,

** *P < 0.01*,

# *P < 0.1. Bold values indicate statistically significant*.

## Discussion

In this study, we found that circulating irisin concentrations were not associated with habitual self-reported PA or ST among children. Moreover, irisin concentrations were negatively associated with BMI, BMI z-score, WC, DBP, and triglyceride levels among children with low PA levels, and negatively related to fasting blood glucose among children with high PA levels. As for ST stratifications, irisin concentrations were negatively associated with BMI and fasting blood glucose only among children with high ST status. Additionally, our study provided new evidence that there was a significant interaction between PA and irisin concentrations on BMI, BMI z-score, and DBP.

Some studies had questioned the regulatory role of PA on circulating irisin concentrations in humans. In the present study, we aimed to provide evidence of associations between habitual PA as well as ST and circulating irisin concentrations. Our results were in agreement with the findings which reported a non-significant association of PA with circulating irisin concentrations (18, 24–26). Various researches found an increase in irisin concentrations after intensive exercise (17, 24, 26). However, the acute exercise-induced increase in irisin concentrations seemed to be a transient phenomenon. Irisin concentrations were up-regulated by prolonged aerobic exercise at a moderate intensity, but were no longer elevated by 90 min and significantly lowered by 20 min of recovery (17). Moreover, another study aimed to explore detailed time-course changes in the irisin response to acute exercise, and suggested that irisin concentrations declined to the baseline at 6 h after exercise (16). In addition, a 6-month randomized trail suggested that irisin was stable within-person with high long-term reliability (27). Accordingly, possible explanations for the uncorrelation between habitual PA and irisin concentrations are as follows. Firstly, exercise could induce a transient irisin elevation but irisin concentrations tend to decline to the stable baseline soon after exercise. In this study, we did not measure participants' irisin concentrations immediately after PA. Secondly, only a short bout of PA is necessary to mobilize irisin from its precursor and the further increase will only occur when new FNDC5 is recruited to the membrane (26). With regard to ST, previous studies largely defined subjects who did not meet the PA guideline as sedentary individuals but seldom took account into the exact ST levels. However, sedentary behavior is not necessarily the same as a lack of PA. The relationship between ST and irisin concentrations has been under-investigated. Taking different types of ST into consideration, our findings do not support a significant association between ST and circulating irisin concentrations in children. However, further evidence in this area is needed before definite conclusions can be drawn.

As for associations of circulating irisin concentrations with cardiometabolic risk factors, there is no general agreement among different researches. In the current study, irisin concentrations were not associated with any cardiometabolic outcomes, except fasting blood glucose in the overall group, but negatively associated with BMI, BMI z-score, and WC among children with low PA levels. Meanwhile, irisin concentrations were negatively associated with fasting blood glucose levels among children with high PA levels. Previous studies had also reported negative associations of irisin concentrations with markers of adiposity and blood glucose (8, 28). According to the research by Boström et al. (6), irisin had an efficacious effect on the browning of white adipose tissues both in culture and *in vivo* to simulate uncoupling protein 1 (UCP1) expression and a broad program of brown fat-like development. Subsequently, it caused a significant increase in whole-body energy expenditure, and modified susceptibility to weight gain and improved glucose homeostasis. In contrast, some studies showed that circulating irisin concentrations were positively correlated with adiposity indices and fasting blood glucose, putting forward the presumption that irisin concentrations were increased in compensation for the “irisin resistance” among obese or diabetic populations (7, 29, 30). Moreover, some researches failed to detect a relationship between irisin concentrations and adiposity indices as well as blood glucose levels (31, 32). The discrepant findings across several studies in humans may be linked with the differences in study population. In our study, the general population of children were analyzed, which exhibited normal glucose homeostasis and without “irisin resistance.” Additionally, it is interesting that irisin concentrations were negatively associated with BMI, BMI z-score, and WC when adjusting for age and sex. However, the relationship became non-significant after further adjustment for other potential confounders. Further analysis indicated that food intake might have an effect on the magnitude of these associations (data not shown). According to previous research, irisin concentrations might not be affected by food ingestion (11, 25). Still, evidence for the impact of food intake on the magnitude of the relationship between irisin and adiposity indices is insufficient. Further work is needed before definitive conclusions can be made.

Besides, it is noteworthy that we found that the association between irisin concentration and fasting blood glucose was more evident among boys in parallel with girls. The gender-specific influence of irisin in glucose status may be explained by differences in hormones and adipose tissue distribution between genders. Compared with girls, boys might have greater volumes of brown adipose tissue (33).

Here, we also presented an inverse association between irisin concentrations and DBP among the low PA subgroup. Our findings were consistent with an observational study from China (34). Nevertheless, Yan et al. (10) demonstrated a non-significant correlation between circulating irisin concentrations and blood pressure in obese Chinese adults. In view of existing evidence, our knowledge about the association of irisin concentrations with blood pressure in humans is still incomplete. Several experiments were performed to detect the modulating effect of irisin on blood pressure in rats. Zhang et al. (35) suggested that peripheral irisin reduced blood pressure in rats by acting on blood vessels, and that both smooth muscle and endothelial cells were affected by irisin. Another study concluded that administration of irisin decreased blood pressure in spontaneously hypertensive rats by an improvement of endothelial dysfunction of the mesenteric artery through the AMPK-Akt-eNOS-NO signaling pathway (36). However, the biological mechanisms by which irisin regulates blood pressure are not totally unraveled, especially in humans.

A growing body of epidemiological evidence indicates that irisin may have an impact on lipid metabolism. In this study, we found that circulating irisin concentrations were negatively related to triglyceride levels among children with low PA status. This negative association was also presented in other previous research (28, 37). According to the experimental evidence, irisin administration might increase the secretion of glycerol and decrease lipid accumulation by regulating the expression of adipose triglyceride lipase, hormone-sensitive lipase and fatty acid-binding protein 4 (38). Conversely, numerous studies demonstrated a positive relationship between irisin concentrations and lipid metabolism markers (9, 39, 40). Diversities of metabolic and endocrine status among study populations might lead to the inconsistent results (38). Based on the current evidence, little is known about the physiological association between irisin and lipid metabolism in humans. Further research is also needed to elucidate the underlying mechanisms.

Interestingly, we also found significant interactions between circulating irisin concentrations and PA levels on BMI, BMI z-score and DBP. Significantly negative associations of irisin concentrations with BMI, BMI z-score, and DBP were found only among children being inactive. These findings highlighted the possibility that the associations mentioned above were differentially regulated by PA status. Meanwhile, we displayed evidence to support the hypothesis that associations of irisin concentrations with cardiometabolic risk factors among individuals being inactive might raise a compensatory role to counterbalance the increased risk of unhealthy life styles (12). On the other hand, it had been reported that various myokines were secreted and released into circulation induced by exercise (41). Exercise might orchestrate the interplay of various myokines and contribute to benefits for health. We speculate that irisin's interplay with other myokines might counteract its association with cardiometabolic outcomes among subjects being active.

Notably, the present study is the first to evaluate the association of ST levels with circulating irisin concentrations, as well as to provide a further analysis of the interactions between PA and irisin concentrations on cardiometabolic outcomes. However, our study also has some limitations. Firstly, our findings were based on a cross-sectional design, and the causality could not be determined. Secondly, PA and ST levels were subjectively measured using a self-report questionnaire. As compared to accelerometer, questionnaires might underestimate the absolute values of MVPA and ST (42). Nevertheless, the questionnaire was on the basis of the widely used IPAQ and had a moderate reliability and validity according to the pre-investigation. A comparison of IPAQ-SF with accelerometer also indicated that MVPA estimated from IPAQ-SF was moderately correlated with accelerometers (42). Thirdly, the low PA subgroup (MVPA <150 min/week) in our study may only represent those children being typically physically inactive. Fourthly, the acute increases in irisin during exercise might be an important mechanism for exercise-induced improvement in cardiometabolic risk factors. However, we did not able to demonstrate whether irisin is simply a brief intermediary pathway in the relationship between PA and cardiometabolic risk factors, for the reason that we did not conduct blood drawing immediately after exercise. Further research is needed to expound the potential pathways. Moreover, circulating irisin concentrations varied a lot in different studies, which may in part be attributed to the fact that different assay kits were used. In the present study, we used reliable and widely used kits (catalog no. EK-067-29; Phoenix Pharmaceuticals, CA, USA), which were specifically designed to measure circulating irisin concentrations. The irisin concentrations in the current study are comparable with those reported by Thomas et al. (9), who used the same kit in their study. Lastly, we measured irisin concentrations in circulation, but were unable to clarify the detailed source of irisin secretion, which led to difficulties in unraveling the precise associations.

In summary, our study found that there was no association between circulating irisin concentrations and habitual PA or ST levels among children. Furthermore, associations of circulating irisin concentrations with BMI, BMI z-score, and DBP were differentially regulated by PA status. The negative associations of irisin concentrations with BMI, BMI z-score, and DBP were found only among inactive children. These findings imply that irisin may contribute to improvement in cardiovascular system and metabolism, especially among children with unhealthy lifestyles. Future studies are needed to provide a better understanding on the function of irisin and the underlying biological mechanism in humans.

## Data Availability

The datasets for this manuscript are not publicly available. Requests to access the datasets should be directed to YC (chenyj68@mail.sysu.edu.cn).

## Ethics Statement

This project had been approved by the Ethical Committee of Sun Yat-sen University and all the participants and their parents signed the informed consents voluntarily prior to participation in the study.

## Author Contributions

YC and LC designed the study. LC, MT, WT, XZ, NW, and JY contributed to the data collection. LC and MT analyzed the data and drafted the manuscript. YC, SW, JO'R, and FS critically reviewed the manuscript. All authors read and approved the final manuscript.

### Conflict of Interest Statement

The authors declare that the research was conducted in the absence of any commercial or financial relationships that could be construed as a potential conflict of interest.

## Abbreviations

BMI: Body mass index
DBP: Diastolic blood pressure
ELISA: Enzyme-linked immunosorbent assay
FBG: Fasting blood glucose
FNDC5: Fibronectin type III domain containing protein 5
HDL-C: High density lipoprotein-cholesterol
IPAQ: International physical activity questionnaire.
LDL-C: Low density lipoprotein-cholesterol
MPA: Moderate intensity physical activity
MVPA: Vigorous to moderate Intensity physical activity
PA: Physical activity
PGC-1α: PPAR γ coactivator 1 alpha
SBP: Systolic blood pressure
ST: Sedentary time
TC: Total cholesterol
TG: Triglyceride
UCP1: Uncoupling protein1
VPA: Vigorous intensity physical activity
WC: Waist circumference
WHO: World Health Organization
WHtR: Waist height ratio.
