# Supplementary material for: Associations of Circulating Irisin Concentrations With Cardiometabolic Risk Factors Among Children Vary by Physical Activity or Sedentary Time Levels
Source: Front Endocrinol (Lausanne). 2019 Aug 14;10:549. doi: 10.3389/fendo.2019.00549 (PMC6703142; doi:10.3389/fendo.2019.00549)
Supplement: Supplementary file 1 [file Data_Sheet_1.docx]

Table S1 Associations of physical activity and sedentary time with subjects’ irisin concentrations by gender

|  | Boys | |  | Girls | |
| --- | --- | --- | --- | --- | --- |
|  | Coefficient | *P* |  | Coefficient | *P* |
| VPA (min/day) | -0.00053670 | 0.8891 |  | 0.00810 | 0.1063 |
| MPA (min/day) | 0.00390 | 0.2284 |  | -0.00319 | 0.4035 |
| MVPA (min/day) | 0.00170 | 0.4501 |  | 0.00073419 | 0.7844 |
| ST (min/day) | 0.00020002 | 0.8868 |  | -0.00236 | 0.1122 |

VPA, vigorous physical activity; MPA, moderate physical activity; MVPA, moderate-vigorous physical activity; ST, sedentary time.

Models were adjusted for age, parental educational levels, family income and food intake.

Table S2 Associations of subjects’ irisin concentrations with cardiometabolic parameters by gender

|  | Boys | |  | Girls | |
| --- | --- | --- | --- | --- | --- |
|  | Coefficient | *P* |  | Coefficient | *P* |
| Adiposity |  |  |  |  |  |
| BMI (kg/m^2^) | -0.15410 | 0.0626 |  | -0.03142 | 0.6734 |
| BMI z-score | -0.06672 | 0.0707 |  | -0.00355 | 0.9192 |
| WC (cm) | -0.27267 | 0.3075 |  | -0.24269 | 0.2223 |
| WHtR | -0.00082 | 0.6197 |  | -0.00128 | 0.2909 |
| Blood pressure |  |  |  |  |  |
| SBP (mmHg) | -0.10934 | 0.7051 |  | -0.16088 | 0.6316 |
| DBP (mmHg) | -0.26873 | 0.2131 |  | -0.03411 | 0.8721 |
| Glycemic parameters |  |  |  |  |  |
| Fasting glucose (mmol/L) | **-0.03379** | **0.0017** |  | -0.03255 | 0.1752 |
| Lipid parameters |  |  |  |  |  |
| Total cholesterol (mmol/L) | -0.01968 | 0.3089 |  | -0.01002 | 0.6604 |
| Triglyceride (mmol/L) | -0.01528 | 0.0788 |  | 0.00065 | 0.9467 |
| HDL-C (mmol/L) | -0.00485 | 0.5626 |  | 0.00270 | 0.7489 |
| LDL-C (mmol/L) | -0.00564 | 0.7630 |  | -0.01189 | 0.5696 |

BMI, body mass index; WC, waist circumference; WHtR, waist height ratio; SBP, systolic blood pressure; DBP, diastolic blood pressure; HDL-C, high-density lipoprotein; LDL-C, low-density lipoprotein.

Models were adjusted for age, parental educational levels, family income, physical activity (min/day), sedentary time (min/day) and food intake.
